# 

**Published:** 1897-10-16

**Authors:** 


### EARLY EDITION.

**Notices of Births, Deaths, and Marriages** are inserted in this column at a charge of 2s. 6d. for an announcement not exceeding four lines.

### NOTICE TO CORRESPONDENTS.

All MS., Letters, Books for Review, and other matters intended for the Editor should be addressed The Editor, "The Hospital," The Hospital Building, 28 & 29, Southampton Street, Strand, W.C.

The Editor cannot undertake to return rejected MS., even when accompanied by stamped directed envelope.

**Notice to Advertisers and Subscribers.**

All Advertisements, Orders for Copies of The Hospital, and Business Communications should be addressed to **THE MANAGER, (Not to The Editor),** The Hospital, The Hospital Building, 28 & 29, Southampton Street, Strand, London, W.C.

The Cost of Subscription, post free, is as follows :—Per week, 2½d. per quarter, 2s. 8d.; per half-year, 5s. 3d.; per annum, 10s. 6d. Foreign :—Per annum, 15s. 2d.; payable, in all cases, in advance.

### BOOKS RECEIVED.

Red Lion Square Publishing Company.

"Problems in Sociology." By Nap.

Scientific Press.

"Fevers and Infectious Diseases: Their Nursing and Practical Management." By William Harding, M.D., M.R.C.P.

"Manual of Hygiene for Students and Nurses." By John Glaister, M.D., D.P.H.

"Child Life Under Queen Victoria." By Mrs. Finlay Smith.

### SCRAPS AND GLEANINGS.

Meltham has decided to build an isolation hospital for itself.

The new workhouse infirmary at Selly Oak for the poor of King's Norton Union has been formally opened.

About £190 was collected by the Shrewsbury Hospital Saturday Fund this year, an increase of about £14 over 1895.

£105 was collected at Brighton by the Friendly and Trades Societies' Hospital Parade Committee held last month.

A sum of £200 has been voted for the purpose of reflooring the Talbot Ward at the Swansea Hospital with teak tongued and grooved.

### THE STUDENT OF MEDICINE.

**APPOINTMENTS.**

Dr. J. Aherne, appointed Medical Officer for the Gislingham District of the Hartismere Union. J. D. Boyd, F.R.C.P.Edin., Physician to the Deaconess Hospital, Edinburgh. F. E. Corbett-Singleton, L.R.C.P. & S.Edin., appointed Clinical Assistant to Out-Patients at the Chelsea Hospital for Women. R. D. Cran, M.R.C.S., L.R.C.P.Lond., appointed Medical Officer for the Salford District of the Salford Union. Dr. Crutwell, re-appointed Medical Officer for the No. 5 District of the Market Harborough Union. H. C. Dent, M.R.C.S.Eng., L.S.A., appointed Medical Officer for the Northrepps District of the Erpingham Urban. D. K. Draffin, L.R.C.P., L.R.C.S.Irel., appointed Medical Officer for the Troedyrhiw District of the Merthyr Tydfil Union. J. Farrar, M.D.St.And., L.R.C.P., L.R.C.S.Edin., appointed Medical Officer for the Gainsborough District of the Gainsborough Union. Dr. Foulkes, appointed Deputy Medical Officer for the No. 1 District of the Conway Union. D. J. Graham, M.B., C.M.Edin., appointed Assistant Medical Officer to the City Hospital for Infectious Diseases, Edinburgh. T. Harris-Liston, M.R.C.S., &c., appointed Assistant Medical Officer of the Lawn Hospital, Lincoln. G. Hope, D.P.H., L.R.C.P., M.R.C.S., and L.S.A.Lond., appointed Medical Officer of Health for the Greenford Urban District. W. A. Hooton, L.R.C.P.Lond., M.R.C.S., L.D.S.Eng., appointed Dental Surgeon to the Manchester Royal Infirmary. S. C. Jones, M.D.Lond., appointed Medical Officer for the Merthyr Lower Town District of the Merthyr Tydfil Union. J. D. Leigh, M.B., L.R.C.S.Edin., appointed Consulting Surgeon to the Cleveland Cottage Hospital. Amelia M. Le Pelley, M.B.Lond., appointed House Surgeon to the Belgrave Hospital for Children, London, S.W. W. J. McCardie, B.A., M.B., B.C.Cantab., appointed an Anæsthetist to the General Hospital, Birmingham. — Musgrave, M.D.Lond., appointed Medical Officer for the Cromer District of the Erpingham Union. J. Neil, M.D.Aberd., appointed Medical Superintendent to the Warneford Asylum. F. Oliphant, M.B.Edin., appointed House Surgeon to the Kidderminster Infirmary. G. W. Park, M.B., C.M., B.Sc.Edin., appointed Resident Physician at the Middle Ward Isolation Hospital, Motherwell, Lanarkshire. S. Partridge, M.D., L.R.C.S.Edin., appointed Medical Officer of Health to the Darlaston Urban District Council. H. A. Pearson, L.R.C.P., L.R.C.S. appointed Medical Officer for the No. 5 District of the Blandford Union. E. E. Porritt, M.B., C.M.Edin., appointed Assistant Medical Officer to the City Hospital for Infectious Diseases, Edinburgh. J. R. Rolston, L.R.C.P., L.M.Edin., M.R.C.S.Eng., appointed Ophthalmic Surgeon to the Royal Albert Hospital, Devonport. Dr. J. A. Rose, appointed Resident Physician at the Aberdeen City Hospital. J. Selkirk, M.A.Glasg., M.B., C.M., appointed Medical Officer for the Boston Spa District of the Wetherby Union. W. Shirreffs, M.B., C.M.Aberd., appointed Medical Attendant for the Water, Sewerage, and Roads Department of the Aberdeen Town Council. G. W. B. Slader, L.R.C.S., L.M.Irel., L.A.H.Dub., appointed Medical Officer for the Blyton District of the Gainsborough Union.

